# Relationship between Season and Acute Myocardial Infarction, In Iran 

**Published:** 2015-10-27

**Authors:** Abdollah Mohammadian-Hafshejani, Salman Khazaei, Hamid Salehiniya

**Affiliations:** 1 *Department of Epidemiology & Biostatistics, School of Public Health, Isfahan University of Medical Sciences, Isfahan, Iran. Tel: + 98 9139887945. Fax: +98 9139887945. E-mail: amohamadii1361@gmail.com.*; 2 *Department of Epidemiology & Biostatistics, School of Public Health, Hamadan University of Medical Sciences, Hamadan, Iran. Tel: +98 9181501628. Fax: +98 9181501628. E-mail: salman.khazaei61@gmail.com.*; 3 *Minimally Invasive Surgery Research Center, Iran University of Medical Sciences, Tehran, Iran. Tel: +98 9357750428. Fax: +98 2188989123. E-mail:alesaleh70@yahoo.com.*

Cardiovascular disease (CVD) is the most common cause of death in Iran.^1^ There has been an increasing trend in the proportional mortality rate since 1981. In 1995, 47.3% of all deaths were due to CVD. According to the first national burden of disease study for the year 2003, the third highest disability-adjusted life year (DALY) in all ages and both sexes (16% of total burden) was attributed to this disease and it accounted for one billion years of life lost (YLLs) due to premature mortality and 500 thousand years lived with disability (YLDs).^2^ Seasonal variation in admissions and mortality due to acute myocardial infarction (AMI) has been observed in different countries. ^3^^-^^6^  Most studies suggest that the highest and lowest rates of the occurrence of AMI resulting in hospital admission and mortality are observed during winter^5^^-^^7^ and summer,^6^^, ^^7^  respectively. Likewise in Iran, according the results of the author’s study, the lowest hospital admission rate for AMI was seen during summer and the highest during spring and winter: the rates increased by 17.8%, 15.8%, and 4.2% during spring, winter, and autumn, respectively, compared to the summer rate. Also, the highest mortality rate was in winter and the lowest in autumn.^8^ In a study conducted by Vasconcelos et al.^9^ in Portugal, a negative outcome of cold weather conditions on AMI was observed insofar as for every degree decrease in physiological equivalent temperature (PET) in winter, there was an increase of up to 2.2% (95% confidence interval: 0.9% - 3.3%) in day-by-day hospital admission rates. However, different explanations exist for the observed variation in hospital admission and mortality due to AMI in various parts of the world. For instance, Ornato et al.^10^ reviewed the results of other studies in this field and reported that numerous ideas have been offered to clarify the increased prevalence of AMI or its complications in the winter and cold season. A quick conversion in the weather can increase arterial blood pressure, blood viscosity, arterial spasm, plasma fibrinogen and factor VII, serum cholesterol levels, and platelet and red blood cell counts. Exposure to cold weather also has important hemodynamic effects, including a rise in systemic vascular resistance, myocardial oxygen intake, and metabolic rate. Contemporary infections during the winter months, particularly those involving the respiratory tract, have also been claimed as a trigger for acute cardiovascular morbid events. Other mechanisms that have been proposed to describe the increase in cardiovascular events during cold weather include seasonal variation in bodily activity, food, weight, worry and stress in the holiday season, and seasonal variety in the secretion of physiologically active substances similar to those that trigger seasonal depression. Also, Ulmer et al.^11^ concluded that cholesterol, blood pressure, and body mass index were seasonal variants that were significantly higher during winter than in the other seasons in all age groups and in both sexes. In contrast, there is also research that contradicts the aforementioned findings by demonstrating that the majority of AMI cases occur during spring.^12^
